# Self-Expandable Metal Stent in the Management of Malignant Airway Disorders

**DOI:** 10.3389/fmed.2022.902488

**Published:** 2022-07-07

**Authors:** Yang Bai, Ke Zhan, Jing Chi, JinYue Jiang, Shuang Li, Yuting Yin, Yishi Li, Shuliang Guo

**Affiliations:** ^1^Department of Respiratory and Critical Care Medicine, The First Affiliated Hospital of Chongqing Medical University, Chongqing, China; ^2^Department of Gastroenterology, The First Affiliated Hospital of Chongqing Medical University, Chongqing, China; ^3^Department of Gastrointestinal Surgery, Jinshan Hospital, The First Affiliated Hospital of Chongqing Medical University, Chongqing, China; ^4^Department of Respiratory and Critical Care Medicine, Chongqing Shapingba District People's Hospital, Chongqing, China

**Keywords:** central airway obstruction, tracheoesophageal fistula, malignancy, self-expandable metal stent, management

## Abstract

**Background:**

Self-expanding metallic stent (SEMS) is a palliative therapy for patients with malignant central airway obstruction (CAO) or tracheoesophageal fistula (TEF). Despite this, many patients experience death shortly after SEMS placement.

**Aims:**

We aimed to investigate the effect of SEMS on the palliative treatment between malignant CAO and malignant TEF patients and investigate the associated prognostic factors of the 3-month survival.

**Methods:**

We performed a single-center, retrospective study of malignant CAO or TEF patients receiving SEMS placement. Clinical data were collected using the standardized data abstraction forms. Data were analyzed using SPSS 22.0. A two-sided *P*-value <0.05 was statistically significant.

**Results:**

106 malignant patients (82 CAO and 24 TEF) receiving SEMS placement were included. The body mass index (BMI), hemoglobin levels, and albumin levels in the malignant TEF group were lower than in the malignant CAO group (all *P* < 0.05). The procalcitonin levels, C-reactive protein levels, and the proportion of inflammatory lesions were higher in the malignant TEF group than in the malignant CAO group (all *P* < 0.05). The proportion of symptomatic improvement after the SEMS placement was 97.6% in the malignant CAO group, whereas 50.0% in the malignant TEF group, with a significant difference (*P* = 0.000). Three months after SEMS placement, the survival rate at was 67.0%, significantly lower in the malignant TEF group than in the malignant CAO group (45.8% vs. 73.2%, *P* = 0.013). Multivariate analysis revealed that BMI [odds ratio (OR) = 1.841, 95% certificated interval (CI) (1.155-2.935), *P* = 0.010] and neutrophil percentage [OR = 0.936, 95% CI (0.883–0.993), *P* = 0.027] were the independent risk factors for patients who survived three months after SEMS placement.

**Conclusions:**

We observed symptom improvement in malignant CAO and TEF patients after SEMS placement. The survival rate in malignant TEF patients after SEMS placement was low, probably due to aspiration pneumonitis and malnutrition. Therefore, we recommend more aggressive treatment modalities in patients with malignant TEF, such as strong antibiotics, nutrition support, and strategic ventilation. More studies are needed to investigate the prognostic factors in patients with malignant airway disorders receiving SEMS placement.

## Introduction

Malignant central airway obstruction (CAO) and tracheoesophageal fistula (TEF) were the major malignant airway disorders severely affecting the patients' mobility and quality of life (1, 2). Malignant CAO is the obstruction in the trachea and mainstem bronchi due to extrinsic compression or direct invasion of primary lung cancer, metastatic lesions from distant tumors, or anatomically adjacent airway tumors (3–5). Malignant TEF is the pathological channel between the trachea and mainstem bronchi and esophagus due to esophageal tumor or primary lung cancer (6, 7). Patients with malignant CAO or TEF could present with dyspnea, hemoptysis, fever, cough, or pneumonia, resulting in a poor prognosis (3–7). Self-expanding metallic stent (SEMS) is a palliative therapy for malignant CAO and malignant TEF patients, rapidly relieving the symptoms and improving quality of life but not prolonging survival (8–11). Despite this, many malignant CAO or TEF patients continue to experience disease progression and even death within a short period after SEMS placement (12). Therefore, we aim to investigate the effect of SEMS on the palliative treatment of malignant CAO and malignant TEF and the associated prognostic factors.

## Materials and Methods

### Patients

We performed a single-center, retrospective study of malignant CAO or TEF patients who received palliative SEMS placement from July 2013 to March 2021 in the First Affiliated Hospital of Chongqing Medical University in Chongqing, China. The inclusion criteria were: (1) patients diagnosed with malignancy pathologically, (2) chest computed tomography (CT) showing airway obstruction or incompleteness and then bronchoscopy with transbronchial lung biopsy confirming malignant CAO or TEF, (3) the risk of airway re-collapse after the debridement or the purpose of fistula closure, (4) written informed consent for airway intervention including SEMS placement; the exclusion criteria were (1) without cytological or histological confirmation of malignancy, (2) follow-up <1 month, (3) lack of clinical or bronchoscopic information, (4) double stent placement in the esophagus and trachea. Standardized abstraction forms were used to gather information from electronic medical records on demographic features, clinical characteristics, laboratory and radiological findings, airway disorders' details (etiology, location, and degree of stenosis et al.), SEMS shapes, and clinical outcomes. The institutional scientific committee approved the publication of this retrospective study.

### Procedures

Flexible bronchoscopy confirmed the malignant CAO or TEF and visualized the lesion location (upper, middle, or lower third trachea, carina, right main bronchus, and proximal or distal left main bronchus), the stenosis type (intrinsic stenosis, extrinsic stenosis, mixed stenosis), and the stenosis degree (<25%, 25–50, 51–75%, 76–90%, and 90% to complete obstruction in cross-sectional area of the target airway, respectively) (13, 14). The SEMS from Micro-tech Co. Ltd or Boston Scientific Co. Ltd was used in the management of malignant CAO or TEF. The SEMS was accustomed according to the airway 3D reconstruction and the bronchoscopic manifestation. The airway 3D reconstruction at the end of inspiration enabled the diameter detection of the stenosed segment and the adjacent normal airway (15, 16). The SEMS diameter was 2 mm smaller than the average normal diameter of the target airway in patients with malignant CAO; the SEMS diameter was 2 mm greater than the average normal diameter of the target airway in patients with malignant TEF, reducing the possibility of stent migration. The target airway diameter was measured simultaneously at the proximal and distal ends of the adjacent normal airway. The length of SEMS was 10 mm longer both proximally and distally than the edges of the lesion estimated from bronchoscopy. The type of stent was accustomed according to the location of the disorders and their relationship to the surrounding branch: Y-shaped SEMS for malignant airway disorders involving the carina or main bronchi, and straight SEMS for disorders involving the upper and middle third trachea and distal left main bronchus. Covered or uncovered SEMS was preferred in patients with malignant CAO, and covered SEMS was chosen to seal the fistula in patients with malignant TEF. The covered SEMS was not recommended if it might lay across a patent airway side branch, causing the post-obstructive pneumonitis.

Mechanical debulking with rigid bronchoscopy and tumor ablation with laser, electrocautery, brachytherapy, and cryotherapy were applied under general anesthesia before the SEMS placement. The guidewire was passed via a flexible bronchoscope, and the SEMS delivery device was passed through the guidewire. Then, the SEMS was deployed under direct bronchoscopic observation, achieving a 100% immediate technical success rate. The forceps were used to adjust the SEMS into the desired position by grabbing the proximal suture of the SEMS. Balloon dilatation was employed within the stent to speed the enlarge of the SEMS if necessary.

### Statistical Analysis

Normally distributed data were expressed as mean ± standard deviation ($\overset{¯}{x}$ ± *s*), and a *t*-test or ANOVA was used to compare between groups. Non-normally distributed data were expressed as a median value and interquartile ranges (IQR, 25–75th quartiles), and Mann Whitney test or Kruskal-Wallis test was used to compare between groups. Qualitative data were compared using the Fisher exact test or Chi-squared test. Multivariate logistic regression analysis was performed to determine the risk factors for the 3-month survival in those patients receiving SEMS placement, including the variables that had reached statistical significance in the univariate analysis. We estimated the variance inflation factors to check for multicollinearity before running the multivariate logistic regression analysis. Data were analyzed using SPSS 22.0 statistical software. A two-sided *P*-value <0.05 was considered to be statistically significant.

## Results

### Demographic Features and Clinical Characteristics

We wound consider the SEMS placement if there has been the risk of airway re-collapse after the debridement or the purpose of fistula closure. Patients who did not require SEMS placement were not included in this study. Among 128 patients receiving SEMS placement in our department, a total of 106 patients (73 male and 43 female), including 82 malignant CAO and 24 malignant TEF patients, were finally enrolled according to the above inclusion and exclusion criteria. Twenty two patients were excluded for the following reasons: benign CAO (*n* = 10), benign TEF (*n* = 2), without clinical or bronchoscopic information (*n* = 4), follow-up <1 month (*n* = 6). The demographic features and clinical characteristics of these patients are listed in Table 1. The mean age of these patients was 62.6 ± 8.9 years, median height 1.65 m (range 1.50–1.77 m) and median weight 56.00 kg (range 30.0–90.0 kg), with no significant difference between the malignant CAO and malignant TEF groups. The body mass index (BMI) was significantly lower in the malignant TEF group than in the malignant CAO group (19.00 ± 1.74 vs. 21.61 ± 3.56 kg/m^2^, *P* = 0.013).

**Table 1:** Demographic features and clinical characteristics between malignant CAO and malignant TEF patients.

	**All patients** **(*n =* 106)**	**Malignant CAO patients** **(*n =* 82)**	**Malignant TEF patients** **(*n =* 24)**	**χ^**2**^, *F*, or *Z*-score**	***P*-value**
Gender (male) [*n* (%)]	73 (68.9)	54 (65.9)	19 (79.2)	1.535	0.162
Age (years)	62.6 ± 8.9	62.5 ± 9.1	63.3 ± 8.1	1.141	0.708
Height (m)	1.60 (1.60, 1.70)	1.65 (1.60, 1.70)	168 (1.60, 1.72)	1.247	0.212
Weight (kg)	56.0 (50.0, 65.0)	56.0 (50.0, 68.0)	55.0 (52.0, 59.8)	−1.339	0.181
BMI (kg/m^2^)	21.18 ± 3.33	21.61 ± 3.56	19.71 ± 1.74	11.571	0.013
BMI group (kg/m^2^)					
<18.5	19 (17.9)	15 (18.3)	4 (16.7)	9.911	0.120
18.5–23.9	65 (61.3)	45 (54.9)	20 (83.3)		
24–27.9	20 (18.9)	20 (24.4)	0 (0.0)		
≥28	2 (1.9)	2 (2.4)	0 (0.0)		
Cough [*n* (%)]	60 (56.6)	46 (56.1)	14 (58.3)	0.038	0.518
Hemoptysis [*n* (%)]	17 (16.0)	16 (19.5)	1 (4.2)	3.247	0.060
Chest pain [*n* (%)]	1 (0.9)	1 (1.2)	0 (0.0)	0.295	0.774
Dyspnea [*n* (%)]	78 (73.6)	71 (86.6)	7 (29.2)	31.491	0.000
Choking on water [*n* (%)]	16 (15.1)	2 (2.4)	14 (58.3)	45.259	0.000
Dysphagia [*n* (%)]	12 (11.3)	5 (6.1)	7 (29.2)	9.842	0.005
Smoking [*n* (%)]	66 (62.3)	48 (58.5)	18 (75.0)	2.142	0.109
Tumor resection [*n* (%)]	33 (31.1)	20 (24.4)	13 (54.2)	7.678	0.007
Hypertension [*n* (%)]	32 (30.2)	29 (35.4)	3 (12.5)	4.606	0.042
Diabetes [*n* (%)]	7 (6.6)	5 (6.1)	2 (8.3)	0.150	0.499
CAD [*n* (%)]	6 (5.7)	5 (6.1)	1 (4.2)	0.130	0.589
COPD [*n* (%)]	14 (13.2)	11 (13.4)	3 (12.5)	0.014	0.606
**Pathological diagnosis**					
LUSC [*n* (%)]	32 (30.2)	32 (39.0)	0 (0.0)	29.252	0.000
LUAD [*n* (%)]	13 (12.3)	13 (15.9)	0 (0.0)		
SCLC [*n* (%)]	5 (4.7)	4 (4.9)	1 (4.2)		
ESCC [*n* (%)]	49 (46.2)	27 (32.9)	22 (91.7)		
Others [*n* (%)]	7 (6.6)	6 (7.3)	1 (4.2)		

*CAO, central airway obstruction; TEF, tracheoesophageal fistula; BMI, body mass index; CAD, coronary artery disease; COPD, chronic obstructive pulmonary disease; LUSC, lung squamous cell carcinoma; LUAD, lung adenocarcinoma; SCLC, small cell lung cancer; ESCC, esophageal squamous cell carcinoma*.

The most common symptoms in the malignant CAO group were dyspnea (71/82, 86.6%), cough (46/82, 56.1%), and hemoptysis (16/82, 19.5%), whereas in the malignant TEF group were choking on water (14/24, 58.3%), cough (14/24, 58.3%), dysphagia (7/24, 29.2%), and dyspnea (7/24, 29.2%). Malignant TEF group reported more frequent symptoms of choking on water (58.3% vs. 2.4%, *P* = 0.000) and dysphagia (29.2% vs. 6.1%, *P* = 0.005) than malignant CAO group. The proportion of coexisting hypertension was higher in the malignant CAO group than in the malignant TEF group (35.4% vs. 12.5, *P* = 0.042). The proportion receiving tumor resection surgery was higher in the malignant TEF group than in the malignant CAO group (54.2% vs. 24.4%, *P* = 0.007). The most common pathological type in the malignant TEF group was esophagus squamous cell carcinoma (22/24, 91.7%), and in the malignant CAO group was lung squamous cell carcinoma (32/82, 39.0%), followed by esophagus squamous cell carcinoma (27/82, 32.9%) and lung adenocarcinoma (13/82, 15.9%,), with a significant difference between the pathological types in the malignant TEF and malignant CAO groups (*P* = 0.000).

### Laboratory and Radiological Findings

The Laboratory and radiological findings of these patients are listed in Table 2. The differences in total leukocyte counts and neutrophil ratios between the malignant TEF and malignant CAO groups were not statistically significant (*P* > 0.05). The procalcitonin levels [0.19 (0.05, 1.70) vs. 0.05 (0.05, 0.10) μg/L, *P* = 0.001] and C-reactive protein levels [57.90 (33.10, 82.95) vs. 27.09 (12.20, 52.65) mg/L, *P* = 0.003] were significantly higher in the malignant TEF group than in the malignant CAO group. In contrast, hemoglobin levels [103.5 (95.25, 110.5) vs. 121.00 (112.75, 135.00) g/L, *P* = 0.000] and albumin levels [30.50 (26.25, 35.75) vs. 38.00 (34.00, 41.00) g/L, *P* = 0.000] were significantly lower in the malignant TEF group than in the malignant CAO group. The proportion of positive sputum cultures was significantly higher in the malignant TEF group than in the malignant CAO group (41.7% vs. 11.0%, *P* = 0.002). The etiological agents most frequently isolated in sputum cultures were gram-negative bacilli (*Escherichia coli, Pseudomonas aeruginosa*, and *Acinetobacter baumannii*), followed by *Candida albicans* in these patients (data not shown). The prothrombin time [13.2 (12.4, 19.1) vs. 11.9 (11.4, 12.7) s, *P* = 0.000] and activated partial prothrombin time [28.4 (26.4, 34.0) vs. 25.7 (22.8, 28.6) s, *P* = 0.003] were longer in the malignant TEF group than in the malignant CAO group. The proportion of mediastinal metastases found on chest CT was significantly higher in the malignant CAO group than in the malignant TEF group (65.9% vs. 37.5%, *P* = 0.018). In contrast, the proportion of inflammatory lesions (patchy shadows or solid shadows) found on chest CT was significantly higher in the malignant TEF group than in the malignant CAO group (75.0% vs. 17.1%, *P* = 0.000).

**Table 2:** Laboratory and radiological findings between malignant CAO and malignant TEF patients.

	**All patients(*n =* 106)**	**Malignant CAO patients (*n =* 82)**	**Malignant TEF patients (*n =* 24)**	**χ^**2**^, *F*, or *Z*-score**	***P*-value**
Total leukocyte counts (10^9^/L)	8.14 (6.41, 10.93) (*n =* 105)	8.25 (6.62, 11.25) (*n =* 82)	7.77 (5.03, 8.65) (*n =* 23)	−1.871	0.061
Neutrophil percentage (%)	83.2 (74.8, 90.5) (*n =* 105)	83.0 (75.2, 90.6) (*n =* 82)	86.0 (73.5, 89.2) (*n =* 23)	0.085	0.932
Procalcitonin levels (μg/L)	0.05 (0.05, 0.17) (*n =* 92)	0.05 (0.05, 0.10) (*n =* 72)	0.19 (0.05, 1.70) (*n =* 20)	3.451	0.001
CRP levels (mg/L)	31.3 (16.6, 58.5) (*n =* 95)	27.1 (12.2, 52.7) (*n =* 74)	57.9 (33.1, 82.9) (*n =* 21)	2.941	0.003
Hemoglobin levels (g/L)	117 (107, 130)	121 (113, 135)	104 (95, 111)	−4.909	0.000
Platelet counts (10^9^/L)	260 ± 99	260 ± 95	228 ± 105	0.152	0.068
Albumin levels (g/L)	37 (33, 40)	38 (34, 41)	31 (26, 36)	−4.474	0.000
Prothrombin time (PT) (s)	12.2 (11.4, 13.0)	11.9 (11.4, 12.7)	13.2 (12.4, 19.1)	4.181	0.000
Activated partial PT (s)	26.2 (23.1, 29.4)	25.7 (22.8, 28.6)	28.4 (26.4, 34.0)	2.948	0.003
D-dimer (mg/L)	0.83 (0.42, 1.64) (*n =* 90)	0.83 (0.41, 1.53) (*n =* 68)	0.88 (0.57, 1.84) (*n =* 22)	0.948	0.343
Positive sputum cultures [*n* (%)]	19 (17.9)	9 (11.0)	10 (41.7)	11.887	0.001
Mediastinal metastases [*n* (%)]	63 (59.4)	54 (65.9)	9 (37.5)	6.191	0.018
Inflammatory lesions [*n* (%)]	32 (30.2)	14 (17.1)	18 (75.0)	29.560	0.000

*CAO, central airway obstruction; TEF, tracheoesophageal fistula; CRP, C-reactive protein; PT, prothrombin time*.

### Airway Disorders' Details, SEMS Choices, and Clinical Outcomes

The airway disorders, SEMS choices, and clinical outcomes of these patients are listed in Table 3. The most frequently involved location in the airway was the lower trachea (56/106, 52.8%), followed by proximal left main bronchus (42/106, 39.6%), right main bronchus (41/106, 38.7%), and carina (36/106, 34.0%). The proportion of multiple-location involvement was higher in the malignant CAO group than in the malignant TEF (72.0% vs. 25.0%, *P* = 0.000). The most common stenosis type was extrinsic stenosis (54/106, 50.9%), followed by mixed stenosis (40/106, 37.7%) and intrinsic stenosis (12/106, 11.3%), with a significant difference between stenosis types in the malignant TEF and malignant CAO groups (*P* = 0.000). There was a significant difference in the stenosis degrees between the malignant TEF and malignant CAO groups (*P* = 0.000).

**Table 3:** Airway disorders' details, SEMS choices, and clinical outcomes between malignant CAO and malignant TEF patients.

	**All patients** **(*n =* 106)**	**Malignant CAO patients (*n =* 82)**	**Malignant TEF patients (*n =* 24)**	**χ^2^, *F*, or *Z*-score**	***P*-value**
**Lesion location**					
Upper third trachea [*n* (%)]	17 (16.0)	10 (12.2)	7 (29.2)	3.971	0.052
Middle third trachea [*n* (%)]	34 (32.1)	29 (35.4)	5 (20.8)	1.800	0.220
Lower third trachea [*n* (%)]	56 (52.8)	48 (58.5)	8 (33.3)	4.732	0.037
Carina [*n* (%)]	36 (34.0)	31 (37.8)	5 (20.8)	2.384	0.147
RMB [*n* (%)]	41 (38.7)	36 (43.9)	5 (20.8)	4.166	0.033
Proximal LMB [*n* (%)]	42 (39.6)	36 (43.9)	6 (25.0)	2.773	0.075
Distal LMB [*n* (%)]	12 (11.3)	8 (9.8)	4 (16.7)	0.883	0.272
Multiple location [*n* (%)]	65 (61.3)	59 (72.0)	6 (25.0)	17.225	0.000
**Stenosis type**					
Intrinsic stenosis [*n* (%)]	12 (11.3)	1 (1.2)	11 (45.8)	30.357	0.000
Extrinsic stenosis [*n* (%)]	54 (50.9)	48 (58.5)	6 (25.0)		
Mixed stenosis [*n* (%)]	40 (37.7)	33 (40.2)	7 (29.2)		
**Stenosis degree**					
<25% [*n* (%)]	13 (12.3)	0 (0.0)	13 (54.2)	60.091	0.000
25-50% [*n* (%)]	4 (3.8)	0 (0.0)	4 (16.7)		
51-75% [*n* (%)]	46 (43.4)	42 (51.2)	4 (16.7)		
76-90% [*n* (%)]	39 (36.8)	37 (45.1)	2 (8.3)		
91-100% [*n* (%)]	4 (3.8)	3 (3.7)	1 (4.2)		
**SEMS shape**					
Straight stent [*n* (%)]	68 (64.2)	57 (69.5)	11 (41.7)	4.526	0.033
Y-shaped stent [*n* (%)]	38 (35.9)	25 (30.5)	13 (54.2)		
Interval between admission and SEMS placement (days)	8 (4, 14)	6 (3, 12)	14 (10, 18)	3.672	0.000
Symptomatic improvement after SEMS insertion [*n* (%)]	92 (86.8)	80 (97.6)	12 (50.0)	36.637	0.000
3-month survival [*n* (%)]	71 (67.0)	60 (73.2)	11 (45.8)	6.274	0.013

*CAO, central airway obstruction; TEF, tracheoesophageal fistula; RMB, right main bronchus; LMB, left main bronchus; SEMS, self-expandable metal stent*.

The proportion of Y-shaped SEMS (54.2% vs. 30.5%) in the malignant TEF group was higher than in the malignant CAO group. The interval between admission and SEMS placement was significantly longer in the malignant TEF group than in the malignant CAO group [14 (10,18) vs. 6 (3, 12) days, *P* = 0.000)]. The symptomatic improvement was seen in 92 out of 106 patients immediately after the SEMS placement. The symptomatic improvement rate after SEMS implantation was higher in the malignant CAO group than in the malignant TEF group (97.6% vs. 50.0%, *p* = 0.000). The 3-month survival rate was lower in the malignant TEF group than in the malignant CAO group (45.8% vs. 73.2%, *P* = 0.013).

### Multivariate Analysis of Risk Factors for the 3-Month Survival

We notice some malignant CAO or TEF patients continue to experience disease progression and even death within a short period after SEMS placement. Therefore, identifying the risk factors contributing to death in patients receiving SEMS placement is essential for the management of malignant airway disorders. A total of 106 patients receiving SEMS in our department were included in this study, of which 35 patients were dead three months after SEMS placement. Table 4 has summarized the comparison of clinical features of those patients who died or survived three months after SEMS placement. The results indicated that dead patients had lower weight, lower BMI, higher neutrophil percentage, a lower proportion of hypertension, a lower proportion of multiple-location involvement, a higher proportion of TEF, a higher proportion of TEF, and a longer duration between admission and SEMS placement than the alive patients (*P* < 0.05).

**Table 4:** The clinical features in patients with malignant airway disorders receiving SEMS placement in which significant differences were found when the patients were divided in two groups (alive or dead three months later).

	**ALL patients** **(*n =* 106)**	**Alive at follow-up** **(*n =* 71)**	**Dead at follow-up** **(*n =* 35)**	**χ^2^, *F*, or *Z*-score**	***P*-value**
Weight (kg)	56.0 (50.0, 65.0)	60.0 (54.0, 68.0)	52.0 (48.0, 58.0)	−3.210	0.001
BMI (kg/m^2^)	21.18 ± 3.33	21.97 ± 3.19	19.59 ± 3.05	2.269	0.000
BMI group (kg/m^2^)					
<18.5	19 (17.9)	8 (11.3)	11 (31.4)	13.824	0.001
18.5–23.9	65 (61.3)	43 (60.6)	22 (62.9)		
24–27.9	20 (18.9)	19 (26.8)	1 (2.9)		
≥28	2 (1.9)	1 (1.4)	1 (2.9)		
Hypertension [*n* (%)]	32 (30.2)	27 (38.0)	5 (14.3)	6.270	0.014
Pathological diagnosis					
LUSC [*n* (%)]	32 (30.2)	29 (40.8)	3 (8.6)	16.343	0.001
LUAD [*n* (%)]	13 (12.3)	6 (8.5)	7 (20.0)		
SCLC [*n* (%)]	5 (4.7)	2 (2.8)	3 (8.6)		
ESCC [*n* (%)]	49 (46.2)	28 (39.4)	21 (60.0)		
Others [*n* (%)]	7 (6.6)	6 (8.5)	1 (2.9)		
Neutrophil percentage (%)	83.2 (74.8, 90.5) (*n =* 105)	81.6 (72.7, 88.3) (*n =* 70)	87.9 (78.7, 93.0) (*n =* 35)	2.476	0.013
Multiple-location involvement [*n* (%)]	65 (61.3)	49 (69.0)	16 (45.7)	5.366	0.033
The reason for SEMS					
CAO [*n* (%)]	82 (77.4)	60 (84.5)	22 (62.9)	6.274	0.013
TEF [*n* (%)]	24 (22.6)	11 (15.5)	13 (37.1)		
Interval between admission and SEMS placement (days)	8 (4, 14)	6 (3, 12)	14 (10, 18)	3.672	0.000

*SEMS, self-expandable metal stent; BMI, body mass index; LUSC, lung squamous cell carcinoma; LUAD, lung adenocarcinoma; SCLC, small cell lung cancer; ESCC, esophageal squamous cell carcinoma; CAO, central airway obstruction; TEF, tracheoesophageal fistula*.

Univariate analysis indicated that weight, BMI, hypertension, neutrophil percentage, multiple-location involvement, and the reason for SEMS (CAO or TEF) were potential risk factors for patients who died three months after SEMS placement (*P* < 0.05). The variance inflation factor was always lower than 10 for these potential risk factors, confirming the weak collinearity. Multivariate analysis revealed that BMI [odds ratio (OR) = 1.841, 95% certificated interval (CI) (1.155–2.935), *P* = 0.010] and neutrophil percentage [OR = 0.936, 95% CI (0.883–0.993), *P* = 0.027] were the independent risk factors for patients who survived three months after SEMS placement, suggesting that the prognosis of patients with malignant airway disorders receiving SEMS placement was associated with malnutrition and infection (Table 5).

**Table 5:** Univariate and multivariate analyses of potential risk factors for the survival in malignant airway diseases at 3 months after SEMS placement.

	**Univariate analysis**		**Multivariate analysis**	
	**OR (95% CI)**	***P*-value**	**OR (95% CI)**	***P*-value**
Weight (kg)	1.058 (1.012–1.106)	0.014	0.886 (0.775–1.014)	0.078
BMI (kg/m^2^)	1.287 (1.095–1.514)	0.001	1.841 (1.155–2.935)	0.010
Hypertension [*n* (%)]	3.111 (1.067 −9.068)	0.016	1.847 (0.052–6.796)	0.356
Neutrophil percentage (%)	0.950 (0.907–0.995)	0.028	0.936 (0.883–0.993)	0.027
Multiple–location involvement [*n* (%)]	2.479 (1.057–5.817)	0.037	2.395 (0.716–8.006)	0.156
The reason for SEMS, TEF [*n* (%)]	0.362 (0.141–0.930)	0.035	0.984 (0.250–3.868)	0.982
Interval between admission and SEMS placement (days)	0.965 (0.908–1.025)	0.245	–	–

*SEMS, self-expandable metal stent; OR, odd ratio; CI, certificated interval; BMI, body mass index; TEF, tracheoesophageal fistula*.

## Discussion

Malignant CAO and malignant TEF were the major malignant airway disorders severely affecting the patients' mobility and quality of life. In this study, we retrospectively analyzed the demographic features, clinical characteristics, laboratory and radiological findings, airway disorders' details, SEMS choices, and clinical outcomes. We also identified the BMI and reason for SEMS (CAO or TEF) as risk factors for the 3-month survival in these patients receiving SEMS placement. The malignant CAO should be considered in malignant patients presenting with dyspnea, dry cough, and hemoptysis, and the malignant TEF in malignant patients presenting with choking on water, dysphagia, and cough, especially in those with a BMI <24kg/m^2^ after the resection surgery of esophagus squamous cell carcinoma. In this study, patients with malignant TEF were often associated with pneumonitis (elevated procalcitonin and C-reactive protein levels, and chest CT showing typical signs of pneumonitis) and malnutrition (decreased hemoglobin and albumin levels), probably leading to a lower survival rate than patients with malignant CAO. Bronchoscopy is the gold standard for the diagnosis of malignant CAO and TEF, determining the pathological types and malignant airway disorders' details while avoiding the risk of worsening the aspiration pneumonitis with repeated gastroscopy in the malignant TEF (2).

SEMS is the palliative treatment to improve mobility and quality of life in patients with malignant CAO or TEF if the radical surgery is contraindicated given the patients' poor clinical status (respiratory distress, pneumonitis, malnutrition) and advanced-stage (stage III–IV) malignancy. SEMS is meth-waved by a shape memory nickel-titanium alloy with substantial elastic formation at a transition temperature of 30°C. The elastic formation of the SEMS permits the easy compression into its delivery device; the shape memory of SEMS allows the resistance to radially compressive forces during coughing; the mesh structure of SEMS enables the conformation to complex and asymmetric lesions in the airway (17). The type, length, diameter, and shape of the SEMS are determined according to the airway 3D reconstruction and the bronchoscopic manifestation. In this study, the deployment of SEMS was observed using bronchoscopy, ensuring a 100% immediate technical success rate and the 97.6% symptom improvement rate in the malignant CAO group and 50% in the malignant TEF group, which was consistent with other studies. In one of the most extensive series (82 patients with CAO, 50 had lung cancer), symptomatic improvement occurred in 87.8% of patients receiving SEMS placement (12). Breitenbücher and colleagues reported a 100% immediate technical success rate and a 100% symptom improvement rate in complex malignant CAO patients (18). In patients with malignant TEF receiving covered SEMS in the tracheobronchial or esophageal under sedatives or general anesthesia, the symptom improvement rate was 80% (19).

Patients with malignant TEF require the covered SEMS to seal the fistula, resulting in a longer interval between admission and SEMS placement in the malignant TEF group than in the malignant CAO group, as shown in this study. During this interval, more aggressive treatment modalities are needed to improve clinical outcomes and survival in patients with malignant TEF, such as strong antibiotics, nutrition support, and strategic ventilation. Gram-negative bacilli (*Escherichia coli, Pseudomonas aeruginosa*, and *Acinetobacter baumannii*) and *Candida albicans* were commonly isolated in the sputum culture or bronchoalveolar lavage fluid in these malignant patients (20, 21). We must note that multi-resistant *Acinetobacter baumannii* infection is associated with a higher risk of death (22). Broad-spectrum cephalosporins (ceftazidime or cefepime), β-lactam/β-lactamase inhibitor combinations (tazobactam or sulbactam), or carbapenems (meropenem or imipenem) can be used empirically in the initial phase, and if necessary, in combination with antifungal drugs. Targeted antibiotics are guided by microbiological culture and drug sensitivity test results. Rigid bronchoscopy with jet ventilation is required in malignant TEF patients with severe respiratory failure during SEMS placement. Strategic ventilation with lower pause pressure and peak inspiratory pressure permits sufficient oxygenation and carbon dioxide evacuation and reduces the risk of subsequent regurgitation of gastric secretions through the TEF into the lungs, leading to the worsening of aspiration pneumonitis (23, 24). Percutaneous endoscopic feeding tube or gastrojejunal feeding tube for enteric feeding and artificial nutrition support is recommended in patients with malignant patients as we noticed that lower BMI was associated a poor survival in the multivariate analysis (25).

There were some limitations that should be addressed in this study. The patients included in the study were from the First Affiliated Hospital of Chongqing Medical University with certain spatial restrictions. The sample size was small relative to the number of independent variables that might be entered in our multivariate logistic regression, leading to the multivariate logistic regression analysis bias (26). We did not have enough data regarding cancer staging and therapies analysis before SEMS placement in patients with malignant airway disorders. Most patients were not followed up, and this study did not provide bronchoscopic observation of SEMS-associated complications. We did not have enough data to calculate what symptoms improved and what symptoms remained among malignant TEF patients after SEMS placement, either the causes of death. Large-scale prospective studies are needed to investigate the prognostic factors in patients with malignant airway disorders receiving SEMS placement.

## Conclusion

We retrospectively analyzed the malignant CAO or TEF patients receiving SEMS placement at the First Affiliated Hospital of Chongqing Medical University, none of which were candidates for surgical treatment. SEMS placement improved symptoms in most malignant CAO patients, whereas in some malignant TEF patients. The survival rate in malignant TEF patients after SEMS placement was low, probably due to malnutrition and infection. Therefore, we recommend more aggressive treatment modalities to improve clinical outcomes and survival in patients with malignant TEF, such as strong antibiotics, nutrition support, and strategic ventilation, especially in those with a BMI <24kg/m^2^ after the resection surgery of the esophagus squamous cell carcinoma. More studies are needed to investigate the prognostic factors in patients with malignant airway disorders receiving SEMS placement.

## Data Availability Statement

The original contributions presented in the study are included in the article/Supplementary Material, further inquiries can be directed to the corresponding author/s.

## Ethics Statement

Written informed consent was obtained from the individual(s) for the publication of any potentially identifiable images or data included in this article.

## Author Contributions

SG and YL designed the study. YB, KZ, and JC performed the research and analyzed the data. YB, JC, KZ, and JJ were involved in data discussion, drafting, and editing the manuscript. All authors contributed to the article and approved the submitted version.

## Funding

The present study was funded by the Chongqing Science and Health Joint Medical Research Project (2020FYYX222) and Chongqing Medical University Future Medical Young Innovation Team Development Support Program (W0119).

## Conflict of Interest

The authors declare that the research was conducted in the absence of any commercial or financial relationships that could be construed as a potential conflict of interest.

## Publisher's Note

All claims expressed in this article are solely those of the authors and do not necessarily represent those of their affiliated organizations, or those of the publisher, the editors and the reviewers. Any product that may be evaluated in this article, or claim that may be made by its manufacturer, is not guaranteed or endorsed by the publisher.
